# Space-Time Clustering of Childhood Leukemia: Evidence of an Association with ETV6-RUNX1 (TEL-AML1) Fusion

**DOI:** 10.1371/journal.pone.0170020

**Published:** 2017-01-27

**Authors:** Christian Kreis, Judith E. Lupatsch, Felix Niggli, Matthias Egger, Claudia E. Kuehni, Ben D. Spycher, on behalf of Swiss Paediatric Oncology Group and the Swiss National Cohort Study Group

**Affiliations:** 1 Institute of Social and Preventive Medicine (ISPM), University of Bern, Bern, Switzerland; 2 University Children's Hospital Zurich, Zurich, Switzerland; National Health Research Institutes, TAIWAN

## Abstract

**Background:**

Many studies have observed space-time clustering of childhood leukemia (CL) yet few have attempted to elicit etiological clues from such clustering. We recently reported space-time clustering of CL around birth, and now aim to generate etiological hypotheses by comparing clustered and nonclustered cases. We also investigated whether the clustering resulted from many small aggregations of cases or from a few larger clusters.

**Methods:**

We identified cases of persons born and diagnosed between 1985 and 2014 at age 0–15 years from the Swiss Childhood Cancer Registry. We determined spatial and temporal lags that maximized evidence of clustering based on the Knox test and classified cases born within these lags from another case as clustered. Using logistic regression adjusted for child population density, we determined whether clustering status was associated with age at diagnosis, immunophenotype, cytogenetic subtype, perinatal and socioeconomic characteristics, and pollution sources.

**Results:**

Analyses included 1,282 cases of which 242 were clustered (born within 1 km and 2 years from another case). Of all investigated characteristics only the t(12;21)(p13;q22) translocation (resulting in ETV6-RUNX1 fusion) differed significantly in prevalence between clustered and nonclustered cases (40% and 25%, respectively; adjusted OR 2.54 [1.52–4.23]; p = 0.003). Spatio-temporal clustering was driven by an excess of aggregations of two or three children rather than by a few large clusters.

**Conclusion:**

Our findings suggest ETV6-RUNX1 is associated with space-time clustering of CL and are consistent with an infection interacting with that oncogene in early life leading to clinical leukemia.

## Introduction

The etiology of childhood leukemia remains largely unknown. Multiple factors including genetic susceptibility, exogenous and endogenous exposures, and chance are thought to play a role [1]. Except for ionizing radiation in high doses, no environmental risk factors have been established [2, 3]. The rarity of the disease and the existence of biologically distinct subtypes with potentially diverse etiologies complicate the search for causal pathways [1]. Several chromosomal abnormalities are observed with increased frequency among cases of childhood leukemia (cytogenetic subtypes) [1, 4]. These are considered to be disease initiating events mostly occurring *in utero* or in early life, which are followed by further genetic alterations that then lead to overt disease [5]. Environmental triggers might be involved in both initial and subsequent mutations.

Several hypotheses regarding a possible infectious etiology of CL have been proposed. Kinlen’s [6, 7] population mixing hypothesis suggests that childhood leukemia is an aberrant response to some yet unidentified infectious agent and that this infection might explain localized increases in incidence following rapid population inflows in rural areas. Smith [8] proposed that the characteristic childhood peak of acute lymphoblastic leukemia (ALL)—observed in more affluent societies at ages 2–6 years for precursor B-cell ALL—might be the result of an infection that occurred *in utero*. The delayed infection hypothesis proposed by Greaves [9] posits a two-hit model: a first hit occurring *in utero* induces genetic alterations in a precursor B-cell, while the second hit precipitating the outbreak of overt ALL is consequent to an aberrant immune response to delayed infections following a lack of exposure to common infections in early life.

A number of studies have reported space-time clustering in the incidence of CL providing indirect support for an infectious origin [10–13]. We recently reported evidence of space-time clustering of CL in Switzerland around the time of birth, noting a considerable excess of pairs of cases born <1 km and <2 years apart over the number that would be expected by chance [14]. Unlike most previous studies, our analysis relied on precise geocoded locations of residence of cases, and adjusted for regional population shifts, which can lead to spurious findings of space-time clustering [15].

If space-time clustering of CL is real, the characteristics of cases occurring close to each other compared to more dispersed cases might provide hints about the causes of the clustering and of the disease itself. Morris [16] thus analyzed cases of CL occuring in close proximity in space and time and found weak evidence that a history of measles 2–3 years prior to the onset of disease was more common than among their matched controls. Williams et al. [17] compared clustered and nonclustered cases of leukemia and Hodgkin’s lymphoma in the context of spatial clustering. They observed a greater degree of concordance of common ALL (CD10+, B-cell precursor ALL) among clustered cases than would be expected by chance. To our knowledge, no other study has compared clustered and nonclustered cases of (childhood) leukemia to elicit clues about etiology nor analyzed the propensity of incident cases to cluster depending on cytogenetic subtype. This might partly be due to the difficulty of distinguishing true clusters, i.e. cases occurring close to each other due to a shared etiological factor, from cases occurring close to each other purely by chance. However, a comparison of cases occurring in close proximity to each other—these should include cases from true etiological clusters if such exist—with other, more dispersed cases should still reveal characteristics of true clusters, even though the observed differences with nonclustered cases will be smaller.

The aim of this study was to characterize clustered cases of CL in Switzerland in an effort to generate new hypotheses about the origins of clustering. We defined clustering by proximity in space and time alone, following the approach adopted by Morris [16]. Thus, based on our earlier findings of space-time clustering around the time of birth, a case was defined as clustered if they were born in close spatial and temporal proximity to another case. We investigated whether clustered cases differed from nonclustered cases in terms of diagnostic, demographic, or socioeconomic characteristics or environmental exposures. We also investigated the size of CL clusters to determine whether the clustering resulted from many small aggregations of only two or three children or rather from a few larger clusters.

## Materials and Methods

Ethics approval was granted through the Ethics Committee of the Canton of Bern to the SCCR.

A detailed description of Materials and Methods is given in the S1 Appendix.

### Population

The study population included all children recorded in the Swiss Childhood Cancer Registry (SCCR) who were born in Switzerland and diagnosed with leukemia at age 0–15 years between 1 January 1985 and 31 December 2014—a sample 22% larger than that of our previous analysis. The SCCR is a population-based registry of all childhood cancers diagnosed in Switzerland with an estimated coverage of 91% during the study period and of about 95% since 1995 [18]. Geocoded residential addresses of cases at the time of birth were obtained from the SCCR. We inspected all cases living <50 m apart for possible sibling relationships and retained only one case from any identified sibling pair. Data on clinical characteristics were obtained from the SCCR in a similar manner. Cytogenetic data became available in 1994 when the earliest karyotype tests were carried out; since 1999 both karyotype and fluorescence in situ hybridization (FISH) analyses have been performed routinely. We also linked SCCR cases with corresponding records in the Swiss National Cohort (SNC) and birth records extracted from the Vital Statistics of the Swiss Federal Office of Statistics using probabilistic record linkage. The SNC is a research platform linking the national censuses with national datasets on birth, mortality, and migration [19]. This allowed us to obtain data on perinatal and socioeconomic characteristics of cases not available directly from the SCCR.

### Characteristics compared between clustered and nonclustered cases

We investigated potential differences in prevalence of the following characteristics between clustered and nonclustered cases:

*Clinical characteristics* included age at diagnosis and the leukemia subtypes acute lymphoid leukemia (ALL) and acute myeloid leukemia (AML). We also distinguished T-precursor ALL and B-precursor ALL separately, and, among the latter, the cytogenetic subtypes ETV6-RUNX1, Philadelphia chromosome, trisomies 4, 10, 17, and high hyperdiploidy (>51–65 chromosomes).

*Demographic and perinatal characteristics* included sex and nationality (Swiss vs. foreign national), extracted from the SCCR; birth weight (<2,500 g; 2,500–4,200 g; >4,200 g), birth order (1st, 2nd, 3rd or later-born) and age of the mother at birth (<25, 25–29, 30–35, >35 years) extracted from national birth records.

*Socioeconomic characteristics* included education level of the household head (compulsory only, upper secondary, tertiary) and household crowding (number of persons per room in tertiles: ≤0.82, 0.83–1.16, ≥1.17) as recorded in the earliest census (1990 or 2000) to which a child could be linked, degree of urbanization of the municipality of residence at birth (urban vs. rural), and neighborhood-based socioeconomic position (neighborhood index of socioeconomic position, Swiss-SEP, in tertiles low, medium, high) [20].

*Environmental exposures* included distance of cases’ residence at birth to the nearest nuclear power plant (NPP; ≤5 km, >5–10 km, >10 km) [21], benzene emitting industrial facility (≤5 km, >5–10 km, >10 km), petrol station (≤100 m, >100–250 m, >250 m), and the nearest highway (≤100 m, >100–500 m, >500 m) [22]. NPP emissions [23], benzene [24], and traffic exhaust [25] are discussed as potential risk factors for childhood leukemia. These particular factors were considered because temporal variations in emissions could, assuming a causal relationship, potentially lead to temporal and localized increases in leukemia risk.

### Statistical analysis

In a first step, we assessed space-time clustering of cases of CL for place and time of birth using the Knox test [26], following the same procedure as in our previous analysis [14]. This test counts the number of pairs of cases that lie in close spatial *and* temporal proximity of each other, i.e. closer than a prespecified spatial and temporal lag, and assesses whether it exceeds the number that is expected by chance. We computed Knox tests for a range of spatial and temporal lags and selected the combination showing the strongest evidence of space-time clustering as critical lags. Tests were carried out using a Monte Carlo procedure that accounts for uneven shifts of the residential population [15]. We used Baker’s max method to calculate a p-value adjusted for multiple testing over different combinations of spatial and temporal lags [27]. We classified a case of CL as clustered if the child was born within the critical lags of at least one other case or otherwise as nonclustered.

In a second step, we ran logistic regressions with clustering status as dependent variable to assess associations with the characteristics listed above. Associations were tested using likelihood ratio tests. Since we applied the critical lags uniformly across the study area, in a densely populated area and in the absence of space-time clustering a case is more likely to be close to another case by chance alone. A characteristic might thus be associated with clustered cases not because it affects the propensity of clustering but because it correlates with population density. In separate regression models, we therefore adjusted for an index of child population density. This index represents the log odds of a case being clustered rather than nonclustered by chance alone, i.e. of another case occurring nearby in time and space. Moreover, for characteristics that are themselves geographically determined (e.g., proximity to a pollution source) inference from logistic regression models is not valid because a dependency between cases is induced [17]. For these variables we therefore calculated p-values through Monte-Carlo simulation. Finally, we used Holm’s [28] procedure to adjust the p-values for multiple testing of different characteristics.

In the last step, we used graph methods [29] to link all clustered cases of CL into individual local clusters. A cluster is thus defined as a group of cases in which each case lies within the critical spatial and temporal lag of at least one other case. After linking all close cases of CL we tabulated the clusters thus identified by the number of children they comprise. Then, for any given cluster size we calculated the probability that the number of clusters with this many children or more in the empirical sample would occur by chance, i.e. in the absence of a tendency of cases to cluster, using Monte Carlo simulations.

A detailed description of Materials and Methods is given in the S1 Appendix.

## Results

### Study population

**Fig 1:**
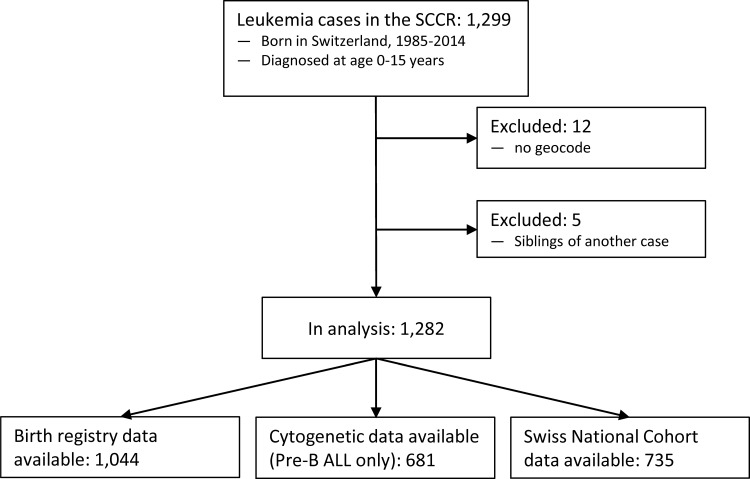
Flow chart of study population.

We identified 1,299 eligible cases of CL in the SCCR. After excluding 12 cases because of missing geocodes and 5 cases due to a sibling relationship with another case, 1,282 cases were included in the analysis (Fig 1). Diagnostic information (age at diagnosis, leukemia subtype) and characteristics related to place of residence (urbanization, neighborhood SEP, environmental exposures) were available for all 1,282 cases. Information on perinatal and socioeconomic characteristics as well as the cytogenetic subtype (collected only from 1994 onward) was available only for subsets of cases due to incomplete data and linkages (Fig 1).

### Space-time clustering

Knox tests showed the strongest evidence of space-time clustering for a spatial lag of <1 km and a temporal lag of <2 years (Table 1). For these threshold values, we observed 152 close pairs of cases of leukemia, an excess of 19% over the 128 close pairs expected under the null hypothesis of no clustering (Monte Carlo P = 0.014). The p-value adjusted for the multiple testing over the 20 different combinations of spatial and temporal lags was 0.12. We thus classified cases of childhood leukemia as clustered if their residence at birth and date of birth was <1 km and <2 years apart from another case. Of the 1,282 cases, 242 (19%) were clustered.

**Table 1:** Results of Knox tests of cases of leukemia at place and time of birth of children 0–15 years of age.

	* *	Months			
		6	12	18	24
** **	*Obs*.*/Exp*.	8/11.5	24/22.9	38/34.2	54/45.2
**0.5 km**	*Ratio*	0.69	1.05	1.11	1.19
** **	*p-value*	0.903	0.464	0.317	0.136
	*Obs*.*/Exp*.	30/32.5	67/64.5	110/96.4	152/127.6
**1 km**	*Ratio*	0.92	1.04	1.14	1.19
** **	*p-value*	0.692	0.387	0.078	0.014
	*Obs*.*/Exp*.	71/83.5	156/165.5	260/247.5	346/327.7
**2 km**	*Ratio*	0.85	0.94	1.05	1.06
** **	*p-value*	0.914	0.738	0.182	0.136
	*Obs*.*/Exp*.	276/307.7	564/610.3	912/912.4	1174/1208.2
**5 km**	*Ratio*	0.90	0.92	1.00	0.97
** **	*p-value*	0.958	0.956	0.415	0.779
	*Obs*.*/Exp*.	686/730.3	1414/1448.3	2153/2165.3	2827/2867.1
**10 km**	*Ratio*	0.94	0.98	0.99	0.99
** **	*p-value*	0.929	0.762	0.497	0.656

Obs., number of observed close pairs; Exp., number of expected close pairs. P-values calculated using a Monte Carlo procedure adjusting for regional population shifts [15].

### Comparison of clustered and nonclustered cases

Results of the analyses of the association between clinical and perinatal characteristics of cases of CL and their propensity to cluster in space-time are presented in Table 2. The only characteristic that showed an association with clustering status independent of child population density was the ETV6-RUNX1 subtype. Among the 569 cases that had been cytogenetically tested for this subtype, 37 out of 93 (40%) clustered cases were carriers of the translocation, compared to only 118 out of 476 (25%) nonclustered cases (unadjusted odds ratio [OR] 2.00, 95% CI 1.26–3.19). This association became stronger in the regression model adjusting for child population density (OR 2.54, 95% CI 1.52–4.23) and remained statistically significant after correcting for multiple testing using Holm's correction (P = 0.0027) [28]. By contrast, none of the other cytogenetic subtypes showed any clear evidence of an association with clustering status nor did any of the clinical and perinatal attributes (Table 2).

**Table 2:** Comparison of clinical and perinatal attributes between clustered* and nonclustered cases of CL both unadjusted and adjusted for local child population density.

		Clustered Cases		Nonclustered Cases		Unadjusted			Child Density Adjusted^a^		
Characteristics		N = 242		N = 1040							
		n/N	%	n/N	%	OR	CI	p	OR	CI	p
Age at diagnosis (years)	0	10 /242	(4.1)	56 /1040	(5.4)	0.80	(0.40–1.61)	0.468	0.86	(0.40–1.87)	0.349
	1–5	142 /242	(58.7)	638 /1040	(61.3)	1.00			1.00		
	6–10	59 /242	(24.4)	209 /1040	(20.1)	1.27	(0.90–1.78)		1.35	(0.93–1.96)	
	11–15	31 /242	(12.8)	137 /1040	(13.2)	1.02	(0.66–1.56)		0.90	(0.56–1.44)	
Immunophenotype											
ALL		202 /242	(83.5)	830 /1040	(79.8)	1.28	(0.88–1.85)	0.188	1.27	(0.85–1.90)	0.246
B-cell ALL		168 /242	(69.4)	725 /1040	(69.7)	0.99	(0.73–1.34)	0.930	1.05	(0.75–1.46)	0.789
Cytogenetic subtype											
ETV6-RUNX1		37 /93	(39.8)	118 /476	(24.8)	2.00	(1.26–3.19)	0.004	2.54	(1.52–4.23)	<0.001
Philadelphia		1 /42	(2.4)	4 /304	(1.3)	1.83	(0.20–16.77)	0.615	0.85	(0.09–8.47)	0.889
High Hyperdiploidy		34 /109	(31.2)	183 /538	(34.0)	0.88	(0.56–1.37)	0.567	0.77	(0.48–1.25)	0.290
Trisomy 4, 10, 17		15 /79	(19.0)	103 /407	(25.3)	0.69	(0.38–1.27)	0.220	0.61	(0.32–1.16)	0.123
T-cell ALL		21 /242	(8.7)	81 /1040	(7.8)	1.13	(0.68–1.86)	0.649	0.97	(0.56–1.68)	0.921
AML		30 /242	(12.4)	143 /1040	(13.8)	0.89	(0.58–1.35)	0.575	0.82	(0.52–1.30)	0.396
Sex	Male	146 /242	(60.3)	611 /1040	(58.8)	1.07	(0.80–1.42)	0.652	1.15	(0.84–1.57)	0.383
Birth weight (grams)	<2500	8 /203	(3.9)	46 /840	(5.5)	0.71	(0.33–1.52)	0.650	0.77	(0.33–1.77)	0.818
	2500–4200	185 /203	(91.1)	751 /840	(89.4)	1.00			1.00		
	>4200	10 /203	(4.9)	43 /840	(5.1)	0.94	(0.47–1.91)		1.00	(0.46–2.16)	
Birth order	1	94 /188	(50.0)	340 /771	(44.1)	1.00		0.271	1.00		0.891
	2	68 /188	(36.2)	296 /771	(38.4)	0.83	(0.59–1.18)		0.93	(0.63–1.37)	
	>2	26 /188	(13.8)	135 /771	(17.5)	0.70	(0.43–1.12)		1.05	(0.62–1.80)	
Age mother at birth (years)	<25	36 /203	(17.7)	132 /841	(15.7)	1.12	(0.72–1.76)	0.897	1.00	(0.62–1.64)	1.000
	25–29	72 /203	(35.5)	296 /841	(35.2)	1.00			1.00		
	30–35	65 /203	(32.0)	283 /841	(33.7)	0.94	(0.65–1.37)		1.01	(0.67–1.53)	
	>35	30 /203	(14.8)	130 /841	(15.5)	0.95	(0.59–1.52)		1.00	(0.59–1.69)	

Columns two and three indicate the prevalence of each case characteristic among clustered and nonclustered cases in absolute numbers and as percentages. Results of the logistic regressions unadjusted and adjusting for child population density are presented in column four and five, respectively.

* Cases born within 1 km and 2 years from another case.

^a^ For a given case, the child density index reflects the probability of another case occurring within 1 km and 2 years by chance alone (See the S1 Appendix for more details).

Likewise, adjusting for child population density there was no evidence of an association for any of the investigated socioeconomic and environmental characteristics (S1 Table). The apparent association of clustered cases with degree of urbanization was expected due to higher child population density in urban areas and disappeared after adjusting for the latter. Similarly, the apparent associations with foreign nationality—significant in unadjusted analyses after correcting for multiple testing (P = 0.019)—and living ≤250 m from a petrol station were greatly attenuated or reversed after adjusting for child population density.

### Analyses by cluster size

Among the 242 clustered cases of CL we identified a total of 104 individual clusters. The majority of them were small; 82 clusters consisted of just two cases, 16 clusters consisted of three cases, there were four clusters with four cases, and one cluster each with five and nine cases of CL. Table 3 reports the mean, minimum, and maximum number of clusters that occurred in the 999 Monte Carlo data sets under the null hypothesis of no clustering. These data sets on average contained one cluster with five and 0.08 clusters with nine children. The p-values indicate the probability that the number of clusters of a given size or larger observed in the empirical data could have occurred simply by chance. The p-value was smallest for a cluster size of two (P = 0.013) indicating an excess of small clusters in the observed data compared to simulated data. By contrast, the observed number of leukemia clusters of size ≥4 and ≥9 was not statistically significant (P = 0.38 and P = 0.18, respectively), meaning that it was not uncommon for clusters of this size or larger to occur in the Monte Carlo data sets.

**Table 3:** Frequency of individual CL clusters by cluster size in the empirical and 999 Monte Carlo samples.

Size	Observed	Monte Carlo Samples			p-Value
		*Mean*	*Min*	*Max*	
1	1040	1086.522	1022	1148	0.994
2	82	68.936	45	94	0.013
3	16	10.884	2	23	0.073
4	4	2.921	0	11	0.383
5	1	1.088	0	6	0.665
6	0	0.467	0	3	0.688
7	0	0.257	0	3	0.464
8	0	0.131	0	2	0.290
9	1	0.078	0	3	0.181
10	0	0.050	0	2	0.999
11	0	0.023	0	1	0.999
12	0	0.016	0	1	0.999
13	0	0.015	0	1	0.999
14	0	0.008	0	1	0.999
15	0	0.004	0	1	0.999

Size, number of elements in clusters; Observed, number of clusters observed in SCCR data; Monte Carlo Samples, mean, minimum, and maximum number of clusters in the 999 Monte Carlo data sets; p-Value (Monte Carlo), probability of obtaining the observed number of clusters of a given size or bigger in the Monte Carlo samples generated under the null of no clustering. (See S1 Appendix for more details on the sampling of the 999 Monte Carlo data sets).

**Fig 2:**
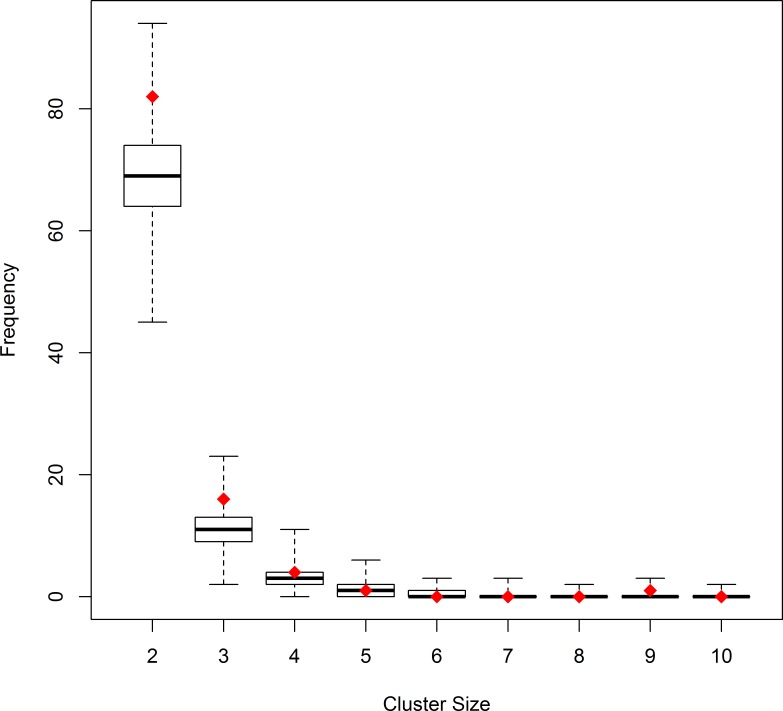
Frequency of clusters of cases of CL by cluster size. Comparison of the frequency of clusters of a given size in the empirical data (red diamonds) with the 999 Monte Carlo data sets generated under the assumption of no space-time clustering (boxplots).

Fig 2 visualizes this observation, highlighting the frequency of clusters of a given size in the empirical data (red diamonds) compared to the Monte Carlo data sets (box plots). The 82 clusters with two children observed in the empirical data lie near the upper end of the range of the corresponding values in the simulated data sets (45–94 clusters). By contrast, for larger cluster sizes of four or more children, the number observed in the empirical data is near the mean number in the simulated data.

## Discussion

### Summary of results

This nationwide study found that the ETV6-RUNX1 gene translocation was more common among CL cases who were born in close spatial and temporal proximity of each other (defined here as clustered) than among more isolated cases. The association between the ETV6-RUNX1 translocation and space-time clustering thus defined became stronger in the analysis adjusting for child population density and remained significant after correcting for multiple testing. The current study also showed that the space-time clustering of CL cases at birth found in an earlier study [14] with overlapping data was primarily due to the numerous small clusters consisting of pairs and triplets of cases rather than to a few larger clusters.

### Our study in the context of the literature

Our results are in accord with previous studies, a majority of which found evidence of clustering of cases of CL around the place and time of birth [11, 30], diagnosis [10, 12, 31–35], or both [13, 16] (studies up to 2004 were reviewed by McNally and Eden [36] and Little [37]). In these studies, the space-time clustering typically was most pronounced for spatial lags of a few kilometers and temporal lags of several months.

Despite this accumulating evidence of the clustering of childhood leukemia, to our knowledge only two previous studies, both from England, have investigated differences between clustered and nonclustered cases to elicit clues about etiology. Looking at 228 cases occurring in the Midlands between 1953 and 1960, Morris observed no significant association between four prenatal events (toxemia, anemia, and X rays of the chest or abdomen) and the tendency of cases to cluster in space and time at birth. However, analyzing the history of six common infectious diseases (measles, pertussis, chicken pox, rubella, mumps, and scarlet fever), Morris observed weak evidence that reports of measles 2–3 years prior to diagnosis were more common among the 18 clustered cases of CL compared to their matched controls, whereas no such difference existed between the 176 nonclustered cases, for which data on infectious histories were available, and controls [16]. In this study, being clustered was defined by proximity in space and time alone as in our study. In a more recent study, Williams et al. found no evidence of association between clustering status of ALL cases and age at diagnosis, immunophenotype, rural vs. urban residence, isolation of area of residence, distance of residence to built-up area, or area-level socioeconomic status [17]. They further investigated whether there was a higher degree of concordance in age at diagnosis, season of diagnosis, and leukemia subtype among cases of the same cluster than would be expected by chance. Evidence of higher concordance was found for common ALL and for a diagnosis in summer. However, Williams et al. only considered spatial clustering and, overall, there was no evidence of a tendency of cases to cluster. The samples of both studies were considerably smaller in size than our sample and they did not have information on cytogenetic subtypes.

The t(12;21)(p13;q22) translocation resulting in the ETV6-RUNX1 fusion gene is the most frequent genetic rearrangement in ALL [38]. In our study, 27.2% of cases with cytogenetic information were carriers. There is convincing evidence from studies of concordant twins showing that this translocation arises *in utero* [39, 40]. It is thought that the translocation is not uncommon in the population at large, yet that further mutations are required for the development of overt leukemia [41]. An early study reported a prevalence of 1% of ETV6-RUNX1 positive cord blood samples from healthy newborns [42]. More recent and larger studies suggested that the prevalence is markedly lower [38, 43]. However, the validity of these findings has been challenged [44].

### Limitations

While the overall sample size in our study was considerable, data on some characteristics were only available for a subset of cases of CL. As a consequence, statistical power was low for some comparisons, particularly for rare gene translocations (such as Philadelphia). Also, we could not perform a separate test of whether space-time clustering is associated with the common ALL subtype (CD10+, B-cell precursor ALL) because the data records in the SCCR do not strictly adhere to a common procedure to differentiate the immunophenotypes of B-cell lineage ALLs. Some socioeconomic and perinatal characteristics could only be obtained through probabilistic record linkage with other databases and may thus include some misclassifications. Furthermore, we classified cases as clustered or nonclustered based solely on proximity using the threshold values that maximized space-time clustering across the entire study area. This definition is likely to have included many pairs of cases occurring close to each other by chance, particularly in more densely populated areas. We therefore adjusted our analyses for child population density by using a measure based on the probability that another case would occur within the critical distance and time lags of a given case in the absence of clustering. Hence such chance clustering should have been accounted for.

### Strengths

The main strength of the study was the availability of precise geocoded residential locations of all cases of CL at birth and for the entire child population in census years. This made it possible to perform space-time clustering analyses at a small spatial scale and to adjust for uneven regional population growth, which may produce spurious evidence of clustering if not controlled for [15]. Exact geocodes of the homes of cases and the child population also allowed us to compute an index of child population density that reflected the probability of another case occurring next to a case simply by chance. Linking the SCCR data with other nationwide routine data sets, we were able to compare clustered and nonclustered cases with regard to a wide array of characteristics that have been hypothesized to play a role in the etiology of childhood leukemia.

### Interpretation of findings

The space-time clustering around the place and time of birth observed in the current study was consistent with the results of our own previous analysis with partly overlapping data [14]. In that paper, which included cases born between 1985 and 2010, we concluded that the scale and timing of clustering might be indicative of an infection occurring *in utero* or shortly after birth. In our previous analysis, stratification of the study sample by age at diagnosis (0–4, 5–15 years) reduced the observed clustering effect, meaning that clustered cases were born close to each other in space and time but diagnosed at widely differing ages [14]. We therefore argued that infections could be involved in early disease initiating events but that the outbreak of overt leukemia required further genetic alterations or environmental stimuli as hypothesized by Greaves [9].

Our current finding that the ETV6-RUNX1 translocation is associated with space-time clustering of CL cases around birth is consistent with the translocation occuring *in utero* [42]. Excesses of cases of CL occurring in spatial and temporal proximity were observed only for small cluster sizes of two or three children and were not driven by a few large clusters. This space-time pattern is consistent with the spreading of common infections that can involve highly localized mini-epidemics [45]. If an infection is indeed causing the clustering, one might speculate about the role of the ETV6-RUNX1 fusion gene. We can think of two potential explanations. First, the infection could cause this translocation. An etiologic role of *in utero* infections has previously been hypothesized for childhood ALL [8]. However, evidence for a direct transforming agent from studies screening for viral sequences in leukemia samples or in neonatal blood spots is still lacking [46]. A second explanation is that, in the presence of the putative infection, the pre-B cells bearing the ETV6-RUNX1 fusion become more susceptible to additional genetic changes leading to uncontrolled replication [39]. Such a process is in line with Greaves’ hypothesis and could explain the tendency of this subtype of ALL to cluster in space and time [9]. This second explanation is more plausible as it is also compatible with different additional genetic changes discernible in ETV6-RUNX1 positive ALLs, even in monozygotic twins [40], and offers a wider timespan for the outbreak of overt leukemia.

The two-year time lag maximizing evidence of space-time clustering appears relatively long considering that a local epidemic might be rather short-lived. However, such a lag is compatible with a brief exposure to an infectious agent paired with an extended age window of susceptibility. The relevant etiological event driving an indvidual space-time cluster might occur concurrently in calendar time–up to several years after birth–but at a different age in each child. If few children move between birth and diagnosis, this will show up as space-time clustering around birth. In our study sample, 34% of children had changed place of residence between birth and diagnosis; however, among children diagnosed before the age of five, only 25% had relocated.

While the space-time pattern of incident cases of CL found in this study thus favors a causal role of an infectious agent, we cannot exclude other environmental factors that might explain the clustering. In fact, the observed lags of <1 km and <2 years maximizing the space-time clustering effect are also compatible with exposure to environmental pollution from local sources with time-varying emission levels. The excesses observed for small cluster sizes would point to numerous small pollutant sources rather than to few large ones. Finally, we cannot rule out that the observed association between the ETV6-RUNX1 translocation and the clustering of cases of CL is due to chance. We investigated numerous factors, none of which had strong prior evidence of being associated with the etiology of CL or with CL clustering. Although we adjusted for multiple testing, this is an exploratory study and validation in independent samples is necessary.

### Conclusions

Our study suggests that the ETV6-RUNX1 translocation is associated with space-time clustering of childhood leukemia around birth. If our findings are confirmed by other studies, future research should investigate a possible link between the ETV6-RUNX1 gene fusion and infections, particularly how infections might induce further genetic alterations in ETV6-RUNX1 positive pre-leukemic clones prompting the outbreak of clinical leukemia.

## Supporting Information

S1 Table Socioeconomic and environmental characteristics. Comparison of prevalence of attributes between clustered and nonclustered cases of CL both unadjusted and adjusted for local child population density.
(DOCX)

S1 Appendix Detailed description of Materials and Methods. (DOCX)

## References

[1] Inaba H, Greaves M, Mullighan CG. Acute lymphoblastic leukaemia. Lancet. 2013;381(9881):1943–55. Epub 2013/03/26. PubMed Central PMCID: PMC3816716. 10.1016/S0140-6736(12)62187-4 23523389 PMC3816716

[2] Wakeford R. The risk of childhood leukaemia following exposure to ionising radiation-a review. Journal of radiological protection: official journal of the Society for Radiological Protection. 2013;33(1):1–25. Epub 2013/01/09. 23296257 10.1088/0952-4746/33/1/1

[3] Wiemels J. Perspectives on the causes of childhood leukemia. Chemico-biological interactions. 2012;196(3):59–67. Epub 2012/02/14. 10.1016/j.cbi.2012.01.007 22326931 PMC3839796

[4] Pui CH, Carroll WL, Meshinchi S, Arceci RJ. Biology, risk stratification, and therapy of pediatric acute leukemias: an update. J Clin Oncol. 2011;29(5):551–65. Epub 2011/01/12. PubMed Central PMCID: PMC3071256. 10.1200/JCO.2010.30.7405 21220611 PMC3071256

[5] Greaves M. Infection, immune responses and the aetiology of childhood leukaemia. Nature reviews Cancer. 2006;6(3):193–203. Epub 2006/02/10. 10.1038/nrc1816 16467884

[6] Kinlen L. Evidence for an infective cause of childhood leukaemia: comparison of a Scottish new town with nuclear reprocessing sites in Britain. Lancet. 1988;2(8624):1323–7. Epub 1988/12/10. 2904050 10.1016/s0140-6736(88)90867-7

[7] Kinlen LJ. An examination, with a meta-analysis, of studies of childhood leukaemia in relation to population mixing. British journal of cancer. 2012;107(7):1163–8. Epub 2012/09/08. 10.1038/bjc.2012.402 22955857 PMC3461174

[8] Smith M. Considerations on a possible viral etiology for B-precursor acute lymphoblastic leukemia of childhood. Journal of immunotherapy. 1997;20(2):89–100. Epub 1997/03/01. 9087381 10.1097/00002371-199703000-00001

[9] Greaves MF. Speculations on the cause of childhood acute lymphoblastic leukemia. Leukemia. 1988;2(2):120–5. Epub 1988/02/01. 3278171

[10] McNally RJ, Alexander FE, Bithell JF. Space-time clustering of childhood cancer in Great Britain: a national study, 1969–1993. International journal of cancer Journal international du cancer. 2006;118(11):2840–6. Epub 2005/12/29. 10.1002/ijc.21726 16381003

[11] McNally RJ, Alexander FE, Birch JM. Space-time clustering analyses of childhood acute lymphoblastic leukaemia by immunophenotype. British journal of cancer. 2002;87(5):513–5. PubMed Central PMCID: PMC489. 10.1038/sj.bjc.6600498 12189547 PMC2376144

[12] Alexander FE, Boyle P, Carli PM, Coebergh JW, Draper GJ, Ekbom A, et al Spatial temporal patterns in childhood leukaemia: further evidence for an infectious origin. EUROCLUS project. British journal of cancer. 1998;77(5):812–7. Epub 1998/03/26. PubMed Central PMCID: PMC2149966. 9514063 10.1038/bjc.1998.132 PMC2149966

[13] Gilman EA, Knox EG. Childhood cancers: space-time distribution in Britain. Journal of epidemiology and community health. 1995;49(2):158–63. Epub 1995/04/01. PubMed Central PMCID: PMC1060101. 7798044 10.1136/jech.49.2.158 PMC1060101

[14] Kreis C, Grotzer M, Hengartner H, Daniel Spycher B, Swiss Paediatric Oncology G, the Swiss National Cohort Study G. Space-time clustering of childhood cancers in Switzerland: A nationwide study. International journal of cancer Journal international du cancer. 2016;138(9):2127–35. Epub 2015/12/10. 10.1002/ijc.29955 26650335

[15] Kulldorff M, Hjalmars U. The Knox method and other tests for space-time interaction. Biometrics. 1999;55(2):544–52. Epub 2001/04/25. 11318212 10.1111/j.0006-341x.1999.00544.x

[16] Morris V. Space-time interactions in childhood cancers. Journal of epidemiology and community health. 1990;44(1):55–8. 2348150 10.1136/jech.44.1.55 PMC1060598

[17] Williams JR, Alexander FE, Cartwright RA, McNally RJQ. Methods for eliciting aetiological clues from geographically clustered cases of disease, with application to leukaemia–lymphoma data. Journal of the Royal Statistical Society: Series A (Statistics in Society). 2001;164(1):49–60.

[18] Schindler M, Mitter V, Bergstraesser E, Gumy-Pause F, Michel G, Kuehni CE, et al Death certificate notifications in the Swiss Childhood Cancer Registry: assessing completeness and registration procedures. Swiss Med Wkly. 2015;145:w14225 10.4414/smw.2015.14225 26700416

[19] Bopp M, Spoerri A, Zwahlen M, Gutzwiller F, Paccaud F, Braun-Fahrlander C, et al Cohort Profile: the Swiss National Cohort—a longitudinal study of 6.8 million people. International journal of epidemiology. 2009;38(2):379–84. Epub 2008/03/11. 10.1093/ije/dyn042 18326512

[20] Panczak R, Galobardes B, Voorpostel M, Spoerri A, Zwahlen M, Egger M, et al A Swiss neighbourhood index of socioeconomic position: development and association with mortality. Journal of epidemiology and community health. 2012;66(12):1129–36. Epub 2012/06/22. 10.1136/jech-2011-200699 22717282 PMC5204371

[21] Spycher BD, Feller M, Zwahlen M, Roosli M, von der Weid NX, Hengartner H, et al Childhood cancer and nuclear power plants in Switzerland: a census-based cohort study. International journal of epidemiology. 2011;40(5):1247–60. Epub 2011/07/14. PubMed Central PMCID: PMC3204210. 10.1093/ije/dyr115 21750009 PMC3204210

[22] Spycher BD, Feller M, Roosli M, Ammann RA, Diezi M, Egger M, et al Childhood cancer and residential exposure to highways: a nationwide cohort study. European journal of epidemiology. 2015;30(12):1263–75. Epub 2015/11/02. 10.1007/s10654-015-0091-9 26520639

[23] Laurier D, Grosche B, Auvinen A, Clavel J, Cobaleda C, Dehos A, et al Childhood leukaemia risks: from unexplained findings near nuclear installations to recommendations for future research. Journal of radiological protection: official journal of the Society for Radiological Protection. 2014;34(3):R53–R68. Epub 2014/06/19. 24938793 10.1088/0952-4746/34/3/R53

[24] Pyatt D, Hays S. A review of the potential association between childhood leukemia and benzene. Chemico-biological interactions. 2010;184(1–2):151–64. Epub 2010/01/14. 10.1016/j.cbi.2010.01.002 20067778

[25] Filippini T, Heck JE, Malagoli C, Giovane CD, Vinceti M. A review and meta-analysis of outdoor air pollution and risk of childhood leukemia. Journal of environmental science and health Part C, Environmental carcinogenesis & ecotoxicology reviews. 2015;33(1):36–66. Epub 2015/03/25. 10.1080/10590501.2015.1002999 PMC4586078 25803195

[26] Knox EG. The Detection of Space-Time Interactions. Roy Stat Soc C-App. 1964;13(1):25–9.

[27] Baker RD. Testing for space-time clusters of unknown size. J Appl Stat. 1996;23(5):543–54.

[28] Holm S. A Simple Sequentially Rejective Multiple Test Procedure. Scandinavian Journal of Statistics. 1979;6(2):65–70.

[29] Bondy JA, Murty USR. Graph Theory. London: Springer; 2008 XII, 655 p.

[30] Gustafsson B, Carstensen J. Evidence of space-time clustering of childhood acute lymphoblastic leukaemia in Sweden. British journal of cancer. 1999;79(3–4):655–7. Epub 1999/02/23. PubMed Central PMCID: PMC2362409. 10.1038/sj.bjc.6690103 10027345 PMC2362409

[31] Birch JM, Alexander FE, Blair V, Eden OB, Taylor GM, McNally RJ. Space-time clustering patterns in childhood leukaemia support a role for infection. British journal of cancer. 2000;82(9):1571–6. 10.1054/bjoc.1999.1072 10789727 PMC2363399

[32] Gilman EA, McNally RJ, Cartwright RA. Space-time clustering of acute lymphoblastic leukaemia in parts of the U.K. (1984–1993). European journal of cancer. 1999;35(1):91–6. 10211094 10.1016/s0959-8049(98)00345-1

[33] Bellec S, Hemon D, Rudant J, Goubin A, Clavel J. Spatial and space-time clustering of childhood acute leukaemia in France from 1990 to 2000: a nationwide study. British journal of cancer. 2006;94(5):763–70. Epub 2006/02/16. PubMed Central PMCID: PMC2374236. 10.1038/sj.bjc.6602980 16479258 PMC2374236

[34] Petridou E, Revinthi K, Alexander FE, Haidas S, Koliouskas D, Kosmidis H, et al Space-time clustering of childhood leukaemia in Greece: evidence supporting a viral aetiology. British journal of cancer. 1996;73(10):1278–83. Epub 1996/05/01. PubMed Central PMCID: PMCPmc2074508. 8630293 10.1038/bjc.1996.245 PMC2074508

[35] Knox EG, Gilman E. Leukaemia clusters in Great Britain. 1. Space-time interactions. Journal of epidemiology and community health. 1992;46(6):566–72. 1494069 10.1136/jech.46.6.566 PMC1059670

[36] McNally RJ, Eden TO. An infectious aetiology for childhood acute leukaemia: a review of the evidence. British journal of haematology. 2004;127(3):243–63. Epub 2004/10/20. 10.1111/j.1365-2141.2004.05166.x 15491284

[37] Little J. Epidemiology of Childhood Cancer. Lyon, France: IARC Scientific Publications No. 149; 1999. 385 p. 10370893

[38] Lausten-Thomsen U, Madsen HO, Vestergaard TR, Hjalgrim H, Nersting J, Schmiegelow K. Prevalence of t(12;21)[ETV6-RUNX1]-positive cells in healthy neonates. Blood. 2011;117(1):186–9. Epub 2010/08/18. 10.1182/blood-2010-05-282764 20713965

[39] Greaves MF, Maia AT, Wiemels JL, Ford AM. Leukemia in twins: lessons in natural history. Blood. 2003;102(7):2321–33. Epub 2003/06/07. 10.1182/blood-2002-12-3817 12791663

[40] Teuffel O, Betts DR, Dettling M, Schaub R, Schafer BW, Niggli FK. Prenatal origin of separate evolution of leukemia in identical twins. Leukemia. 2004;18(10):1624–9. 10.1038/sj.leu.2403462 15356660

[41] Greaves MF, Wiemels J. Origins of chromosome translocations in childhood leukaemia. Nature reviews Cancer. 2003;3(9):639–49. Epub 2003/09/03. 10.1038/nrc1164 12951583

[42] Mori H, Colman SM, Xiao Z, Ford AM, Healy LE, Donaldson C, et al Chromosome translocations and covert leukemic clones are generated during normal fetal development. Proceedings of the National Academy of Sciences of the United States of America. 2002;99(12):8242–7. Epub 2002/06/06. PubMed Central PMCID: PMC123052. 10.1073/pnas.112218799 12048236 PMC123052

[43] Olsen M, Hjalgrim H, Melbye M, Madsen HO, Schmiegelow K. RT-PCR screening for ETV6-RUNX1-positive clones in cord blood from newborns in the Danish National Birth Cohort. J Pediatr Hematol Oncol. 2012;34(4):301–3. Epub 2012/01/06. 10.1097/MPH.0b013e3182332268 22217495

[44] Greaves M, Colman SM, Kearney L, Ford AM. Fusion genes in cord blood. Blood. 2011;117(1):369–70. 10.1182/blood-2010-09-309351 21212294

[45] Onozuka D, Hagihara A. Spatial and Temporal Dynamics of Influenza Outbreaks. Epidemiology. 2008;19(6):824–8. 10.1097/EDE.0b013e3181880eda 18813019

[46] Eden T. Aetiology of childhood leukaemia. Cancer Treat Rev. 2010;36(4):286–97. Epub 2010/03/13. 10.1016/j.ctrv.2010.02.004 20223594

