# Supplementary material for: Space-Time Clustering of Childhood Leukemia: Evidence of an Association with ETV6-RUNX1 (TEL-AML1) Fusion
Source: PLoS One. 2017 Jan 27;12(1):e0170020. doi: 10.1371/journal.pone.0170020 (PMC5271308; doi:10.1371/journal.pone.0170020)
Supplement: S1 Table — Comparison of prevalence of attributes between clustered and nonclustered cases of CL both unadjusted and adjusted for local child population density. (DOCX) [file pone.0170020.s001.docx]

**S1 Table Comparison of socioeconomic and environmental attributes between clustered* and nonclustered cases of CL both unadjusted and adjusted for local child population density**

|  |  | **Clustered Cases** | |  | **Nonclustered Cases** | |  | **Unadjusted** | | |  | **Child Density Adjusted^a^** | | |
| --- | --- | --- | --- | --- | --- | --- | --- | --- | --- | --- | --- | --- | --- | --- |
| **Characteristics** |  | N = 242 | |  | N = 1040 | |  |  |  |  |  |  |  |  |
|  |  | **n/N** | **%** |  | **n/N** | **%** |  | **OR** | **CI** | **p** |  | **OR** | **CI** | **p** |
| **Socioeconomic** |  |  |  |  |  |  |  |  |  |  |  |  |  |  |
| Nationality | Swiss (vs. Foreign) | 170 /222 | (76.6) |  | 846 /982 | (86.2) |  | 0.53 | (0.37 - 0.75) | <0.001 |  | 0.78 | (0.52 - 1.15) | 0.209 |
|  |  |  |  |  |  |  |  |  |  |  |  |  |  |  |
| Education of head of household | compulsory | 20 /153 | (13.1) |  | 74 /564 | (13.1) |  | 1.00 |  | 0.962 |  | 1.00 |  | 0.853 |
|  | upper secondary | 84 /153 | (54.9) |  | 303 /564 | (53.7) |  | 1.03 | (0.59 - 1.78) |  |  | 1.14 | (0.62 - 2.10) |  |
|  | tertiary | 49 /153 | (32.0) |  | 187 /564 | (33.2) |  | 0.97 | (0.54 - 1.74) |  |  | 1.03 | (0.54 - 1.97) |  |
|  |  |  |  |  |  |  |  |  |  |  |  |  |  |  |
| Crowding (No persons/room) in tertiles | 0-0.82 | 53 /157 | (33.8) |  | 242 /578 | (41.9) |  | 1.00 |  | 0.132 |  | 1.00 |  | 0.871 |
|  | 0.83-1.16 | 58 /157 | (36.9) |  | 202 /578 | (34.9) |  | 1.31 | (0.86 - 1.99) |  |  | 1.04 | (0.66 - 1.65) |  |
|  | 1.17-5 | 46 /157 | (29.3) |  | 134 /578 | (23.2) |  | 1.57 | (1.00 - 2.45) |  |  | 1.14 | (0.69 - 1.89) |  |
|  |  |  |  |  |  |  |  |  |  |  |  |  |  |  |
| Urbanicity | Urban (vs. Rural) | 221 /242 | (91.3) |  | 704 /1040 | (67.7) |  | 5.02 |  | 0.514 |  | 1.09 |  | 0.850 |
|  |  |  |  |  |  |  |  |  |  |  |  |  |  |  |
| Neighbourhood SES in tertiles | 1-low | 89 /242 | (36.8) |  | 434 /1040 | (41.7) |  | 1.00 |  |  |  | 1.00 |  |  |
|  | 2-medium | 86 /242 | (35.5) |  | 312 /1040 | (30.0) |  | 1.34 |  | 0.174 |  | 1.21 |  | 0.382 |
|  | 3-high | 67 /242 | (27.7) |  | 294 /1040 | (28.3) |  | 1.11 |  | 0.784 |  | 0.88 |  | 0.607 |
|  |  |  |  |  |  |  |  |  |  |  |  |  |  |  |
| **Environmental** |  |  |  |  |  |  |  |  |  |  |  |  |  |  |
| Distance to NPPs | <=5 km | 3 /242 | (1.2) |  | 13 /1040 | (1.3) |  | 0.99 |  | 0.992 |  | 1.52 |  | 0.816 |
|  | <=10 km | 6 /242 | (2.5) |  | 45 /1040 | (4.3) |  | 0.56 |  | 0.435 |  | 0.79 |  | 0.708 |
| Distance to Cement factories & Refineries | <=5 km | 4 /242 | (1.7) |  | 31 /1040 | (3.0) |  | 0.55 |  | 0.480 |  | 0.64 |  | 0.624 |
|  | <=10 km | 9 /242 | (3.7) |  | 81 /1040 | (7.8) |  | 0.46 |  | 0.225 |  | 0.53 |  | 0.216 |
| Distance to Petrol Stations | <=100 m | 3 /242 | (1.2) |  | 10 /1040 | (1.0) |  | 1.29 |  | 0.821 |  | 1.11 |  | 0.876 |
|  | <=250 m | 26 /242 | (10.7) |  | 54 /1040 | (5.2) |  | 2.08 |  | 0.719 |  | 0.89 |  | 0.646 |
| Distance to highways | <=100 m | 7 /242 | (2.9) |  | 25 /1040 | (2.4) |  | 1.21 |  | 0.803 |  | 1.06 |  | 0.935 |
|  | <=500 m | 42 /242 | (17.4) |  | 174 /1040 | (16.7) |  | 1.05 |  | 0.884 |  | 0.89 |  | 0.691 |

Columns two and three indicate the prevalence of each case characteristic among clustered and nonclustered cases in absolute numbers and as percentages. Results of the logistic regressions unadjusted and adjusting for child population density are presented in column four and five, respectively.

* Cases born within 1 km and 2 years from another case.

^a^ For a given case, the child density index reflects the probability of another case occurring within 1 km and 2 years by chance alone (See the S1 Appendix for more details).
