# Supplementary material for: Space-Time Clustering of Childhood Leukemia: Evidence of an Association with ETV6-RUNX1 (TEL-AML1) Fusion
Source: PLoS One. 2017 Jan 27;12(1):e0170020. doi: 10.1371/journal.pone.0170020 (PMC5271308; doi:10.1371/journal.pone.0170020)
Supplement: S1 Appendix — (DOCX) [file pone.0170020.s002.docx]

**Supplementary material**

**Space-time clustering of childhood leukemia: evidence of an association with TEL-AML1 (ETV6-RUNX1) fusion**

**Christian Kreis^1^, Judith Lupatsch^1^, Felix Niggli^2^, Matthias Egger^1^, Claudia E. Kuehni^1^, Ben D. Spycher^1^ for the Swiss Paediatric Oncology Group and the Swiss National Cohort Study Group**

1. Institute of Social and Preventive Medicine (ISPM), University of Bern, Bern, Switzerland
2. University Children's Hospital Zurich, Zurich, Switzerland

**Corresponding author:**

Ben D. Spycher

Postal address: University of Bern, Institute of Social and Preventive Medicine (ISPM), Finkenhubelweg 11, 3012 Bern, Switzerland

Tel. +41 31 631 33 46

Fax +41 31 631 35 20

Email: [ben.spycher@ispm.unibe.ch](mailto:ben.spycher@ispm.unibe.ch)

# Detailed description of materials and methods

## Population

The study population included leukemia cases recorded in the Swiss Childhood Cancer Registry (SCCR) born and diagnosed in Switzerland at 0-15 years of age between 1985 and 2014. The SCCR is a population-based registry of all childhood and adolescent cancers diagnosed in Switzerland. Estimated average coverage of cases diagnosed at age 0-15 years is 91% over this period; since 1995 coverage has been approximately 95% [[1](#_ENREF_1)]. The SCCR tracks residential histories from diagnosis back to birth [[2](#_ENREF_2)].

## Geocoding

Residential addresses of cases at the time of birth were geocoded to the Swissgrid coordinate system (CH1903 / LV03) by linking them with the database of georeferenced street addresses maintained by Swiss Post (GeoPost), or manually using the geoportal of the Swiss Federal Office of Topography ([www.geo.admin.ch](http://www.geo.admin.ch)). The geocoding process was accurate to within 50 m for 95% of available residential addresses and to <100 m for another 4% of cases; the exact location remained uncertain for less than 1% of addresses (and was geocoded to the center of a town/village). We inspected all cancer cases whose residence coordinates were <50 m apart from each other based on patient record data for possible sibling relationships and retained only one record from any identified sibling pair [[3](#_ENREF_3)]. Data on clinical characteristics were obtained from the SCCR. Collection of results from cytogenetic tests (karyotype, FISH) began in 1994. Perinatal and socioeconomic data not available in the SCCR were obtained through probabilistic record linkage with national birth records and the Swiss National Cohort (SNC), respectively. The SNC is a research platform linking the national censuses with national datasets on mortality and migration [[4](#_ENREF_4),[5](#_ENREF_5)].

## Characteristics compared between clustered and nonclustered cases

We investigated potential differences between clustered and nonclustered cases in the following characteristics:

*Clinical characteristics* included age at diagnosis as well as the leukemia subtypes acute lymphoid leukemia (ALL) and acute myeloid leukemia (AML). We analyzed T-precursor ALL and B-precursor ALL separately, and among the latter distinguished the cytogenetic subtypes ETV6-RUNX1, Philadelphia chromosome, trisomies 4, 10, 17, and high hyperdiploidy (>51-65 chromosomes).

*Demographic and perinatal characteristics* included sex and nationality (Swiss vs. foreign national) both extracted directly from the SCCR; birth weight (<2,500 g, 2,500-4,200 g, >4,200 g), birth order (1^st^, 2^nd^, 3^rd^, or later-born) and age of the mother at birth (<25, 25-29, 30-35, >35 years) as obtained from national birth records.

*Socioeconomic characteristics* included education level of the household head (compulsory only, upper secondary, tertiary), household crowding (number of persons per room in tertiles 0-0.82, 0.83-1.16, 1.17-5) as recorded in the earliest census (1990 or 2000) to which a child could be linked, degree of urbanization of the municipality of residence (urban vs. rural), and neighborhood-based socioeconomic position (neighborhood index of socioeconomic position, Swiss-SEP, in tertiles low, medium, high) [[6](#_ENREF_6)]. The last two variables were gathered for the mother’s residence at the time of birth. Degree of urbanization was based on a typology of municipalities developed by the Federal Statistical Office.

*Environmental exposures* included those known or suspected from the literature to play a role in the etiology of CL, and were considered because temporal variations in emissions might lead to localized increases in leukemia risk. Pollution sources in the analysis included distance of a case's residence at birth to the nearest nuclear power plant (NPP; ≤5 km, >5-10 km, >10 km) [[2](#_ENREF_2)], benzene emitting industrial facility (≤5 km, >5-10 km, >10 km), petrol station (≤100 m, >100-250 m, >250 m), and the nearest highway (≤100 m, >100-500 m, >500 m) [[7](#_ENREF_7)]. Emissions from NPPs [[9](#_ENREF_9)], benzene [[10](#_ENREF_10)], and traffic exhaust [[11](#_ENREF_11)] are discussed as potential risk factors for childhood leukemia. Since 2007 the Swiss Federal Office for the Environment has maintained the Swiss Pollutant Release and Transfer Register (SwissPRTR, <http://www.prtr.admin.ch>), which records facilities that exceed specified emission thresholds for recognized pollutants. For benzene these threshold values are 1000 kg/year for emissions into the atmosphere and 200 kg/year for emissions into water and soil. We included all facilities exceeding one of these limits in any year since 2007: two oil refineries, four cement factories, and one pharmaceutical production site. Geocodes for these sites were obtained from the SwissPRTR. Geocoded locations of petrol stations were obtained from the Federal Statistical Office’s business censuses conducted in 1995, 1998, 2001, 2005, and 2008. Nearest locations were identified for the census year closest to a child’s date of birth. A digital map of the Swiss highway network was obtained from the Federal Office of Spatial Development.

## Statistical Analysis

### Determining critical lags that maximize evidence for space-time clustering

As the first step in the analysis, we assessed space-time clustering of cases of childhood leukemia for place and time of birth using the Knox test [[12](#_ENREF_12)]. This tests whether the number of pairs of cases that lie close to each other in space and time, i.e. closer than a prespecified temporal and spatial lag, exceeds the number of close pairs that is expected by chance. In our earlier study which included cases of CL from 1985 up to 2010, we found strongest evidence for space-time clustering for residence at birth and date of birth using spatial and temporal lags of 1 km and 2 years [[3](#_ENREF_3)]. In this study, we tested spatial lags of 0.5, 1, 2, 5, and 10 km and temporal lags of 0.5, 1, 1.5, and 2 years in accordance with previous studies. The Knox test is known to be susceptible to uneven population shifts, which can lead to spurious positive findings. We therefore accounted for this using a Monte Carlo procedure proposed by Kulldorff and Hjalmars [[13](#_ENREF_13)].

### Monte Carlo sampling

We sampled control locations representative of the geographic distribution of the general child population at the time of cases' birth using a two-step approach (described in detail in [[3](#_ENREF_3)]): first, municipalities were sampled with replacement weighted by the number of male and female births in a case's year of birth. In a second step, we randomly sampled residential locations from the census data (1990, 2000 and 2010) among a case's age group (0-4 years) and sex peers living in the municipalities selected during the first step. To do this we probabilistically sampled residential locations from the two censuses closest to the cases’ year of birth; for a child born in 1994, for instance, we selected a control location with probability 0.6 and 0.4 from the 1990 and 2000 census, respectively.

Mergers and swaps of territory were a common occurrence over the study period. In order to ensure consistent geographical boundaries throughout the study period, we merged neighboring modified municipalities for the purposes of the process of sampling of control locations.

We thus obtained 999 Monte Carlo samples containing potential case locations representative of the study population under the null hypothesis of no clustering with sample sizes equal to the number of cases observed empirically. These datasets were then used to compute standardized counts of close pairs using the expected values and standard errors as proposed by Barton and David [[14](#_ENREF_14)] (We refer to these standardized counts as z-values here). We obtained the p-values for the Knox tests as the number of z-values of the simulated data sets that exceeded the empirical z-value divided by 1,000. We refer to the spatial and temporal lag of the Knox test with the lowest p-value as critical lags. We also calculated a p-value adjusted for multiple testing over different combinations of space-time lags using Baker’s max method [[15](#_ENREF_15)]. Under this method, only the maximum test statistic over all combinations of space-time lags is considered, and the value of the empirical data set ranked against the corresponding values of the simulated data sets.

### Testing for association with clustered cases

Each case of childhood leukemia was subsequently classified as clustered if the child was born in closer spatial and temporal proximity of another case than the critical lags of 1 km and 2 years determined in the first step, or otherwise as nonclustered. We then used clustering status as outcome variable in logistic regression models in order to assess associations with the characteristics listed above (reported as odds ratios with 95% confidence intervals). We used likelihood ratio tests to test whether associations differed from null.

As the critical lags were applied uniformly across the study area, a case living in an area with dense child population was more likely to be close to another case by chance alone (in the absence of space-time clustering). A characteristic might therefore be associated with clustered cases not because it is implicated in the etiology of clustering, but simply because it is associated with population density. We therefore ran separate regression models adjusting for an index of child population density. This index represents the log odds of a case being clustered rather than nonclustered (see section below).

For variables that are geographically determined (degree of urbanization; neighborhood-based socioeconomic position; and distance to NPPs, cement factories and refineries, petrol stations, and highways), the inference from logistic regression is not valid because including such variables induces spatial dependence between cases [[16](#_ENREF_16)]. For instance, if two leukemia cases are close to each other (i.e. clustered) and one of them is close to a pollution source, the likelihood that the other case will also be close to the same source is increased. Conversely, if one of the cases if far from the source, the likelihood that the other one will also be far is increased. This dependency between cases will increase the standard error of the regression coefficient. The standard errors calculated assuming independence of cases will thus underestimate the true errors and lead to erroneous inference. For these situations we therefore calculated p-values through Monte-Carlo simulation by fitting the corresponding regression models to the close pairs in each of the simulated samples and ranking the odds ratios of the empirical sample against those of the Monte Carlo samples. This required computing the geographically determined variables for each of the Monte Carlo samples.

### Index for child population density

Our definition of ‘clustered’ is based on proximity alone and includes proximity of cases that occurs by chance alone rather than through shared aetiological factors. We attempted to correct for this in our regression models by adjusting for an index reflecting the probability that a case becomes ‘clustered’ by chance alone.

Let $X_{i}$ be an indicator variable taking on the value 1 in the event that the $i$^th^ child in the population at risk developes leukaemia and 0 otherwise. Further, let $Y_{i}$ be an indicator variable taking on the value 1 if at least one other child born within given spatial and temporal lags from the $i$^th^ child ( a neighbouring child) develops leukaemia and 0 otherwise. The probability of becoming ‘clustered’ by chance is the conditional probability of $Y_{i}=1$ given that $X_{i}=1$, i.e. $P\left( Y_{i}=1\left| X_{i}=1 \right. \right)$, under the assumption that any two events $X_{i}$ and $X_{j}$ ($i\neq j$) are independent (in our application background incidence rates are allowed to vary over calender time and strictly speaking we therefore assume independence conditional on children’s dates of birth). Under the assumption, $X_{i}$ and $Y_{i}$ are also independent and the probability of interest is simply the unconditional probability $P\left( Y_{i}=1 \right)$. This probability will depend on the background incidence and the number of children at risk in the $i$^th^ child’s neighbourhood. More precisely, let $k_{i}$ denote the number of other children born within the given spatial and temporal lags from the $i$^th^ child (neighbouring children ) and $\bar{p}_{i}$ denote the probability that a given neighbouring child does not develop leukaemia (we assume that this probability is the same for all $k_{i}$ neighbouring children), then, again using the assumption of independent events, $P\left( Y_{i}=1 \right)=1-{\bar{p}_{i}}^{k_{i}}$.

In order to estimate $\bar{p}_{i}$, we first calculated the cancer risk at the national level among children born within the temporal lag (± 2 years) from the $i$^th^ child’s date of birth. This was obtained as the total number of cancer cases in Switzerland with date of birth within this period (based on SCCR data) divided by the total number of live births within this period (based on national birth records). The estimate $\hat{\bar{p}_{i}}$ was calculated as one minus this risk. As an estimate of $k_{i}$, we used the number of children living with the spatial (circle of 1 km radius) around the $i$^th^ child’s place of residence in the census nearest in time to the child’s date of birth.

In order to adjust for the probability of becoming ‘clustered’ by chance in logistic regression models, it is appropriate first to convert this probability to the log-odds scale. Regression models were thus adjusted by including the variable $z_{i}$ defined as follows:

$$z_{i}=\log\left( \frac{\hat{P}\left( Y_{i}=1 \right)}{1-\hat{P}\left( Y_{i}=1 \right)} \right)=\log\left( \frac{1-{\hat{\bar{p}_{i}}}^{\hat{k_{i}}}}{{\hat{\bar{p}_{i}}}^{\hat{k_{i}}}} \right)=\log\left( {\hat{\bar{p}_{i}}}^{\hat{{-k}_{i}}}-1 \right)$$

The appropriateness of this index was demonstrated by applying our definition of being ‘clustered’ to our 999 Monte Carlo samples of potential case locations – in which any clustering is by definition due to chance – and regressing clustered status on this index. If this index does appropriately reflect the probability of becoming clustered by chance, we would expect a regression coefficient close to one. The resulting coefficients from these regressions were indeed very close to one with narrow 95% confidence intervals.

### Adjustment for multiple testing

We used Holm’s procedure to correct the p-values for multiple testing over the different case attributes. This procedure ensures that the familywise error rate (i.e. the probability of making at least one false discovery) is smaller than or equal to the nominal significance level which we set as 0.05. We computed adjusted p-values using the holm function in the R package mutoss.

### Analysis by cluster size

In a final step, we used graph methods [[17](#_ENREF_17)] to combine all close cases into individual clusters and tabulated them by cluster size. Undirected graphs were computed based on the adjacency matrix of the Knox test maximizing space-time clustering. A cluster is thus defined as a group of cases in which each case occurred within the critical spatial and temporal lag of at least one other member of the group. We again applied the Monte Carlo method to calculate the probability under the null hypothesis of no space-time clustering that a given minimal cluster size was reached with the same or greater frequency as in the empirical sample. To this end, we applied the graph method to each of the 999 Monte Carlo samples and ranked the frequency of clusters of a given size or larger observed in the empirical sample against the corresponding frequencies in the Monte Carlo samples. The simulated p-value then is the number of the Monte Carlo samples for which this number exceeded the value for the empirical data divided by 1000. We computed graphs using the graph.adjacency function in the R package igraph.

## References

1. Schindler M, Mitter V, Bergstraesser E, Gumy-Pause F, Michel G, et al. (2015) Death certificate notifications in the Swiss Childhood Cancer Registry: assessing completeness and registration procedures. Swiss Med Wkly 145: w14225.

2. Spycher BD, Feller M, Zwahlen M, Roosli M, von der Weid NX, et al. (2011) Childhood cancer and nuclear power plants in Switzerland: a census-based cohort study. Int J Epidemiol 40: 1247-1260.

3. Kreis C, Grotzer M, Hengartner H, Daniel Spycher B, Swiss Paediatric Oncology G, et al. (2016) Space-time clustering of childhood cancers in Switzerland: A nationwide study. Int J Cancer 138: 2127-2135.

4. Bopp M, Spoerri A, Zwahlen M, Gutzwiller F, Paccaud F, et al. (2009) Cohort Profile: the Swiss National Cohort--a longitudinal study of 6.8 million people. Int J Epidemiol 38: 379-384.

5. Spoerri A, Zwahlen M, Egger M, Bopp M (2010) The Swiss National Cohort: a unique database for national and international researchers. Int J Public Health 55: 239-242.

6. Panczak R, Galobardes B, Voorpostel M, Spoerri A, Zwahlen M, et al. (2012) A Swiss neighbourhood index of socioeconomic position: development and association with mortality. J Epidemiol Community Health 66: 1129-1136.

7. Spycher BD, Feller M, Roosli M, Ammann RA, Diezi M, et al. (2015) Childhood cancer and residential exposure to highways: a nationwide cohort study. Eur J Epidemiol 30: 1263-1275.

8. Wakeford R (2013) The risk of childhood leukaemia following exposure to ionising radiation-a review. J Radiol Prot 33: 1-25.

9. Laurier D, Grosche B, Auvinen A, Clavel J, Cobaleda C, et al. (2014) Childhood leukaemia risks: from unexplained findings near nuclear installations to recommendations for future research. J Radiol Prot 34: R53-R68.

10. Pyatt D, Hays S (2010) A review of the potential association between childhood leukemia and benzene. Chem Biol Interact 184: 151-164.

11. Filippini T, Heck JE, Malagoli C, Giovane CD, Vinceti M (2015) A review and meta-analysis of outdoor air pollution and risk of childhood leukemia. J Environ Sci Health C Environ Carcinog Ecotoxicol Rev 33: 36-66.

12. Knox EG (1964) The Detection of Space-Time Interactions. Roy Stat Soc C-App 13: 25-29.

13. Kulldorff M, Hjalmars U (1999) The Knox method and other tests for space-time interaction. Biometrics 55: 544-552.

14. Barton DE, David FN (1966) The random intersection of two graphs. In: David FN, editor. Research Papers in Statistics (Festschrift for J Neyman). London: John Wiley & Sons. pp. 445-459.

15. Baker RD (1996) Testing for space-time clusters of unknown size. Journal of Applied Statistics 23: 543-554.

16. Williams JR, Alexander FE, Cartwright RA, McNally RJQ (2001) Methods for eliciting aetiological clues from geographically clustered cases of disease, with application to leukaemia–lymphoma data. Journal of the Royal Statistical Society: Series A (Statistics in Society) 164: 49-60.

17. Bondy JA, Murty USR (2008) Graph Theory. London: Springer. XII, 655 p.
